# Unraveling the Mystery of 3-Sulfanylhexan-1-ol: The Evolution of Methodology for the Analysis of Precursors to 3-Sulfanylhexan-1-ol in Wine

**DOI:** 10.3390/foods11142050

**Published:** 2022-07-11

**Authors:** Jennifer R. Muhl, Lisa I. Pilkington, Bruno Fedrizzi, Rebecca C. Deed

**Affiliations:** 1 School of Chemical Sciences, Waipapa Taumata Rau, The University of Auckland, Private Bag 92019, Auckland 1142, New Zealand; jmuh198@aucklanduni.ac.nz (J.R.M.); lisa.pilkington@auckland.ac.nz (L.I.P.); b.fedrizzi@auckland.ac.nz (B.F.); 2 School of Biological Sciences, Waipapa Taumata Rau, The University of Auckland, Private Bag 92019, Auckland 1142, New Zealand

**Keywords:** aroma precursors, calibration methods, grape wines, liquid chromatography–mass spectrometry, sample preparation, 3-sulfanylhexan-1-ol

## Abstract

Volatile polyfunctional thiol compounds, particularly 3-sulfanylhexan-1-ol (3SH) and 3-sulfanylhexyl acetate (3SHA), are key odorants contributing to the aroma profile of many wine styles, generally imparting tropical grapefruit and passionfruit aromas. 3SH and 3SHA are present in negligible concentrations in the grape berry, juice, and must, suggesting that they are released from non-volatile precursors present in the grape. The exploration of the nature and biogenesis of these precursors to 3SH and 3SHA has proven important for the elucidation of polyfunctional thiol biogenesis during alcoholic fermentation. The development and validation of appropriate analytical techniques for the analysis of 3SH precursors in enological matrices have been extensive, and this review explores the analysis and discovery of these precursor compounds. The development of analytical methods to analyze 3SH precursors, from the selection of the analytical instrument, sample preparation, and methods for standardization, will first be discussed, before highlighting how these techniques have been used in the elucidation of the biogenesis of 3SH and 3SHA in grape wines. Lastly, the future of thiol precursor analysis will be considered, with the development of new methods that greatly reduce the sample preparation time and enable multiple precursors, and the thiols themselves, to be quantitated using a single method.

## 1. Introduction

**Figure 1:**
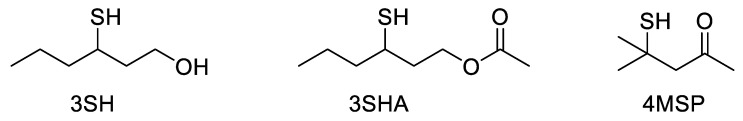
The three key volatile thiols in wine: 3-sulfanylhexan-1-ol (3SH), 3-sulfanylhexylacetate (3SHA), and 4-methyl-4-sulfanylpentan-2-one (4MSP).

Wine aroma is the result of complex interactions between the components of the wine matrix. Although a large proportion of volatile aroma compounds are present only in trace amounts (e.g., ng L^−1^), many of these compounds have a high sensory impact and further influence the perception of each other by enhancing, suppressing, or adding to the overall aroma profile. To identify and quantify these trace aroma compounds, they first need to be separated from the complex wine matrix. This requirement has led to the uptake of chromatographic systems coupled to various detectors for their subsequent analysis. In the quest to characterize wine aroma compounds, the most appropriate detector, in the first instance, is olfaction. Volatile polyfunctional thiol compounds, including 3-sulfanylhexan-1-ol (3SH), 3-sulfanylhexylacetate (3SHA), and 4-methyl-4-sulfanylpentan-2-one (4MSP) (Figure 1), were first identified in wines using a gas chromatography–olfactometry (GC-O) instrument [1,2]. The comparison of retention times and fragmentation patterns using gas chromatography–mass spectrometry (GC-MS) allowed the identification of the compounds involved in the aroma zones identified by GC-O [1,2], and the investigation into the role of polyfunctional thiols in wine aroma began.

**Figure 2:**
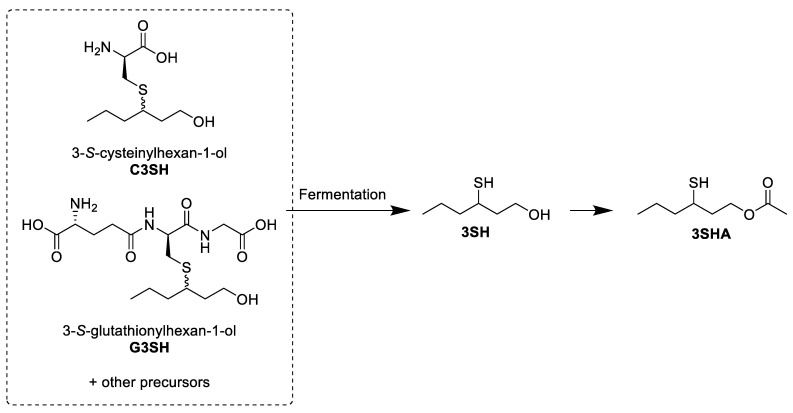
3SH is released from non-volatile precursors, including 3-S-cysteinylhexan-1-ol (C3SH) and 3-S-glutathionylhexan-1-ol (G3SH), during fermentation, and 3SH can then be acetylated via yeast to 3SHA.

Over the past 26 years, since the first identification of 4MSP, extensive research has been directed toward the investigation of these volatile thiols. The impact of 3SH, 3SHA, and 4MSP on wine aroma can be explained by their incredibly low perception thresholds, even in wine matrices (60 ng L^−1^ [3], 4.2 ng L^−1^ [2], and 3 ng L^−1^, respectively) [1]. In addition to their role as impact odorants in the distinctive aroma of Sauvignon blanc wines [2,3,4,5,6], volatile thiols have been shown to be important in many white wines, including Chardonnay, Colombard, Gewürztraminer, Petit Manseng, and Riesling, with 3SH being most prevalent. Volatile thiols also feature in many botrytized white wine styles and wines made from red grape varieties [7,8,9,10,11,12,13,14,15,16,17]. Despite their importance in wine aroma, there is still a lack of understanding of volatile thiol formation. 3SH, 3SHA, and 4MSP are not present in the grape must, which has led to an extensive range of studies to elucidate their biogenesis and release during alcoholic fermentation, with yeast playing a vital role [18,19,20,21]. The discovery of the non-volatile cysteine conjugate precursor of 3SH, 3-S-cysteinylhexan-1-ol (C3SH) (Figure 2) [22], and other volatile thiols, including 4MSP, prompted the further exploration of these precursors along with other pathways to 3SH formation. These studies have required several analytical tools. Through harnessing analytical techniques and instruments, as they became commercially available and more widespread through the scientific community, several methods for quantifying precursors to 3SH have been developed. Although the analytical approaches to the detection and quantification of volatile thiols have been reviewed before [23], this did not extend to their origins, and the further development of methods has also since been reported.

In this review, we explore the analysis and discovery of the precursors to volatile thiols, with a focus on 3SH and 3SHA. This review will give an overview of the development of analytical methods to analyze 3SH precursors, from the selection of the analytical instrument, sample preparation, and standardization methods. From here, the application of these methods to the elucidation of 3SH biogenesis and identification of new precursors will be discussed, followed by a discussion of the future of thiol precursor analysis.

## 2. Analytical Methods to Analyze Wine Aroma Precursors

### 2.1. Early Exploration of 3SH Precursors: Choosing an Analytical Instrument

**Scheme 1:**
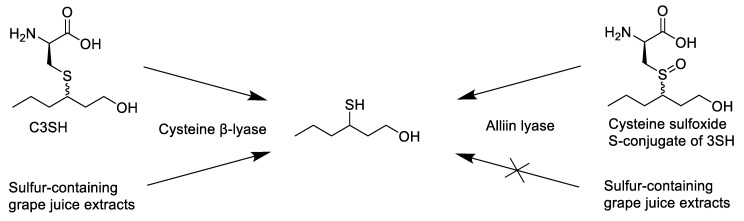
Simplified visual representation of the work of Tominaga et al. [22], elucidating the nature of the cysteine conjugate precursor by an enzyme assay.

The first methods used for the exploration of potential precursors to 3SH and 3SHA used GC-MS instruments. Even before the first formal identification of 3SH in Sauvignon blanc wine [1], it was noted that volatile thiols were not present in the grape must, but that the fermentation of a model medium supplemented with Sauvignon blanc must extracts was able to release 4MSP, a closely related compound [24]. The first precursors to 3SH to be identified were the cysteine conjugates to the thiol (both diastereomers) (Figure 2), which had previously been proposed as precursors to similar sulfur-containing compounds in other plants, such as cabbage and onions [25,26]. Given that cysteine is a biological source of sulfur [27], it was hypothesized that this could provide the thiol moiety of these volatile thiols. As there are a number of cysteine sulfoxide conjugates that can release thiols when metabolized, these also needed to be investigated as potential precursors [22,25,28]. The cysteine S-conjugates (C3SH and cysteine sulfoxide S-conjugates) of the target thiols were identified as one group of precursors to 3SH and 3SHA by comparison of the products of synthetic compounds and grape extracts after incubation with a cysteine β-lyase or alliin lyase, an enzyme specific for the cleavage of cysteine sulfoxide S-conjugates (Scheme 1) [22].

### 2.2. Gas Chromatography–Mass Spectrometry in 3SH Precursor Analysis

These early explorations of 3SH biogenesis involved both direct and indirect analysis of C3SH, or, more accurately, precursors of 3SH, as it had not been shown that C3SH was the only precursor measured using indirect methods. Indirect strategies depended on the enzymatic release of the volatile thiols from must extracts using established methods to measure the original thiol precursor concentrations [22,29,30,31]. The use of enzymes brought with it several challenges, not least the variation in enzyme activity leading to variability between batches [29]. To mitigate enzyme variability, an internal standard (IS) was used to give an indication of enzyme activity and allow the conversion of the thiol precursors to be compared with the conversion of the IS. However, it was suggested, in early work, that accurate quantification could not be obtained without stable isotopically labeled IS compounds due to the complexity of the matrix and the dangers of assuming that similar compounds will behave in the same way during sample preparation and analysis [32]. The need to utilize a stable-isotope-labeled IS also dictated the requirement to use a mass spectrometer as the detector. Direct approaches mitigating the variability in sample preparation were also developed, improving the detection of the cysteinylated thiol precursors by GC-MS. To analyze molecules via GC-MS, the analytes must be volatile at the raised temperatures used in GC analysis, (around 200 to 350 °C) and be thermally stable [33]. To analyze non-volatile precursors of 3SH, the use of GC-MS required chemical derivatization to volatilize the target analyte. Unlike the indirect approaches that were initially explored, the derivatization of the cysteinylated precursors permitted the direct detection of these compounds, albeit with labor-intensive sample preparation [34]. The typical procedure for the direct analysis of C3SH using derivatization and GC-MS required only 500 µL of sample from which the cysteinylated precursors were isolated on an immobilized copper column before derivatization [22,34,35]. The derivatization step was optimized, and both silylation and perfluoro-acylation were evaluated before heptafluro-acylation (HFA) was ultimately selected as the derivatization method of choice. This method was chosen, as the HF derivatives of the C3SH diastereomers could be chromatographically separated [35]. The determination of the diastereomeric ratio of C3SH is of particular interest, since the R and S enantiomers of 3SH have different perception thresholds (50 ng L^−1^ and 60 ng L^−1^) and differ slightly in their aroma descriptors [36].

**Scheme 2:**
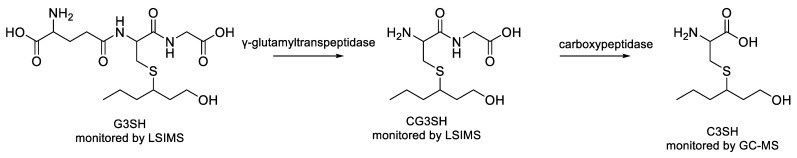
Metabolism of G3SH to C3SH by enzymes (adapted from Peyrot des Gachons et al. [29]).

The discovery of other 3SH precursors, specifically the glutathionylated precursor, led to a decline in the popularity of the GC-MS analysis of cysteinylated thiol precursors [31]. Unlike C3SH, 3-S-glutathionylhexan-1-ol (G3SH) could not be volatilized sufficiently for analysis by GC-MS, and the wider availability of LC-MS technology by the early 2000s made the LC-MS analysis of these precursors more viable. The first analysis of glutathionylated precursors to 3SH was performed via indirect methods involving the analysis of the C3SH released when crude sulfur-containing extracts from the grape must were treated with γ-glutamyltranspeptidase [31]. Peyrot des Gachons et al. [31] found that grape must samples treated with γ-glutamyltranspeptidase released C3SH, which could be analyzed by GC-MS, and cysteinylglycine-3SH (CG3SH), which could be analyzed by liquid secondary-ion mass spectrometry (LSIMS). LSIMS technology was also used to identify G3SH in the samples by comparison with synthetic standards of G3SH and monitor the decrease in the G3SH concentration after treating with γ-glutamyltranspeptidase [31]. This work by Peyrot des Gachons et al. [31] was the first to identify a glutathionylated thiol precursor in the grape must, which would later be identified as one of the most important classes of thiol precursors. However, the technology available at the time limited the potential for further research. LSIMS depended on samples being purified sufficiently to allow the detection of the target analytes, as they were not coupled to a chromatographic system to reduce matrix interference. Additionally, the authors noted that the γ-glutamyltranspeptidase used to metabolize G3SH to CG3SH was likely contaminated with carboxypeptidase, which was responsible for C3SH being released along with CG3SH (Scheme 2).

### 2.3. Initial 3SH Precursor Analysis with Liquid Chromatography–Mass Spectrometry

It was not until 2008 that LC systems coupled with MS detectors began to be utilized for the analysis of C3SH and other thiol precursors [37], but, from then, they became the standard method. The main advantage of LC-MS for thiol precursor analysis was the reduction in sample preparation required before analysis. The sensitivity of the methods can be compared by looking at the parameters reported for the method validation.

The limit of detection (LOD) and limit of quantification (LOQ) are commonly reported measures of method sensitivity based on the signal (S)-to-noise (N) ratio of the method. The definition typically used by the wine research community is that the LOD is the concentration at which the analyte can be reliably detected in the sample (S/N = 3), and the LOQ is the concentration at which the analyte can be reliably quantified in the sample (S/N = 10). Indeed, the LOD and LOQ for optimized GC-MS and LC-MS/MS analyses of C3SH are comparable (Table 1), despite the significantly fewer steps in sample preparation for LC-MS methods (Table 2) [35,38,39,40,41].

## 3. Development of LC-MS Methods for 3SH Precursor Analysis

### 3.1. Sample Preparation

Unlike GC-MS systems, LC-MS systems can analyze complex aqueous matrices, such as grape juice and wine, sometimes without sample treatment or purification before injection. Having said this, it can be advantageous to conduct some sample preparation to reduce noise and improve the LOD and LOQ of the analytes [47], in addition to protecting the system from exposure to matrix components, which may compromise the analysis. Many different sample preparation methods have been utilized throughout the literature. Some are as simple as filtering the sample prior to injection, to avoid damaging the HPLC column [42,43,45], while others involve more extensive purification of the target analytes from the rest of the matrix [37,40,41,42,44]. Unsurprisingly, sample preparation methods are significantly influenced by the sample matrix. Even within the field of thiol biogenesis in wine, there are a range of matrices to be studied, from the grape berries and other solid plant matter to the grape juice, must, and wine. Each poses its own challenges, with its own major components of the matrix to remove before analysis. A summary of the sample matrix and preparation methods from previous studies analyzing thiol precursors is shown in Table 2.

When analyzing thiol precursors in solid samples, for example, grape skins, seeds, leaves, or commercially available tannins, the precursors need to be extracted into a liquid phase before analysis [46,48,49]. When developing methods involving extraction from solid matrices, the major challenge is optimizing the extraction solvent and conditions to maximize the extraction of the target analytes, standardizing the procedure so that these analytes can be quantified [49]. For thiol precursors in liquid matrices, such as grape juice or wine, further purification is typically required before analysis. The high concentrations of non-target compounds, such as glucose and fructose in grape juice and sweet wine styles, pose both technical and analytical challenges [47]. Such compounds can be insoluble in the mobile phases, may damage the LC column, and can lead to deposits in key components of the system, particularly the MS source. Further, their variation in composition between each sample can lead to significant matrix effects, where the co-elution of non-volatile or non-target compounds leads to the suppression or enhancement of ionization, reducing the reliability of the method and worsening the sensitivity [47]. Sample preparation can strongly influence the reliability of the method and should be standardized within a given method to ensure consistency in the extraction of the target analytes. However, the data available in the literature for analytical methods targeting thiol precursors do not suggest that there is a significant advantage in extensive sample preparation. Although solid-phase extraction (SPE) is a simple method for extracting the target analytes from many matrix components that may interfere with analysis, effectively simplifying and standardizing the sample matrix, it does not offer a significant improvement in method sensitivity (Table 3). One reason for this is that the solid phase used during SPE for the analysis of these thiol precursors is often substantially similar to the solid phase of the LC column, which would mean that the compounds extracted along with the target analytes are those that will behave similarly in the LC system and are thus likely to co-elute and interfere with the signal [50]. Several sample preparation techniques and analytical instruments can be used for the analysis of thiol precursors, but these do not seem to correlate well with the LOD and LOQ values reported (Table 2). Instead, the method used for measuring LOD and LOQ, and the matrix used for this validation, have more impact on the reported accuracy of the analytical methods.

### 3.2. Choice of Calibration Method

To accurately measure the concentrations of the target compounds, a calibration must be conducted. The earliest attempt to quantify volatile thiol concentrations in wines utilized GC-O and involved sequential dilution until the perception threshold of the compound was reached [32]. However, this technique is not feasible for quantifying the non-volatile precursors to these compounds. Even then, the authors noted that the reliable quantitation of compounds would best be achieved using a MS detector and a stable-isotope-labeled analogue of the target compound [32]. Unfortunately, stable-isotope-labeled compounds tend to be expensive and are often commercially unavailable, meaning that researchers must synthesize them, adding both complexity and cost to the process. Since these early studies, four main quantification techniques, i.e., external calibration (EC), standard addition (SA), internal standard calibration (ISC), and the stable isotope dilution assay (SIDA), have been used to quantify 3SH precursors (Table 4).

#### 3.2.1. External Calibration

EC is the simplest calibration method. The choice of the matrix for the calibration solutions is important, as different matrices can influence the responses significantly [51]. Kobayashi et al. [39] prepared their calibration solutions in a blank aqueous matrix, which resulted in a very good LOD and LOQ. However, these metrics do not accurately reflect the performance of the method, only the instrument’s performance. This was reflected in the recovery being less than 100% when the analytes were spiked into real samples [39]. Thus, the LOD and LOQ are not useful for comparing the method reported by Kobayashi et al. [39] to other methods, as the choice of calibration matrix could have artificially improved the performance. Not only are LC-MS systems susceptible to matrix effects, the potential losses of analytes during sample preparation should be accounted for where possible [47,51]. Creating a matrix-matched calibration curve can address some of the issues with matrix effects, but matrix matching is challenging when a large number of samples, with subtly different compositions, are being analyzed [51]. Mattivi et al. [42] sought to address these issues by using grape juice and wine as the matrix for the calibration curve; however, sample matrices can still have subtle differences in composition from the calibration matrix. While these methods using EC have comparable sensitivity compared with methods using alternative calibrations (Table 4), the accuracy could be in question due to potential matrix interference [39,42].

**Table 4:** A summary of recent quantitative methods for the analysis of 3SH precursors.

Authors	Year	Quantitation Method ^b^	Analytical Instrument	C3SH			G3SH			CG3SH			GC3SH		
				IS	LOD (ppb)	LOQ (ppb)	IS	LOD (ppb)	LOQ (ppb)	IS	LOD (ppb)	LOQ (ppb)	IS	LOD (ppb)	LOQ (ppb)
Thibon et al. [35]	2008	ISC	GC-ITMS	S-Benzyl cysteine	0.33	1.1	-	-	-	-	-	-	-	-	-
Luisier et al. [37]	2008	SIDA	APCI-HPLC-MS in neg ion mode	*d_2_*-C3SH	3	10	-	-	-	-	-	-	-	-	-
Thibon et al. [38]	2010	ISC	GC-MS	S-Benzyl cysteine	0.022	0.088	-	-		-	-	-	-	-	-
Roland et al. [41]	2010	SIDA	nLC-MS/MS	*d_2_*-C3SH	0.31	1.05	*d_2_*-G3SH	0.061	0.20	-	-	-	-	-	-
Roland et al. [52]	2010	SIDA	nLC-MS/MS	*d_2/3_*-C3SH ^d^	0.20	0.95	*d_2/3_*-G3SH ^d^	0.059	0.32	-	-	-	-	-	-
Kobayashi et al. [39]	2010	EC	HPLC-MS/MS, GC-MS	-	0.044	0.13	-	0.041	0.12	-	-	-	-	-	-
Capone et al. [40]	2010	SIDA	LC-MS/MS	*d_8_*-C3SH	0.04	0.12	*d_9_*-G3SH	0.13	0.39	-	-	-	-	-	-
Capone et al. [53]	2011	ISC^c^	HPLC-MS/MS	-	-	-	-	-	-	*d_8_*-C3SH	0.2	0.5	-	-	-
Mattivi et al. [42]	2012	EC	UPLC-MS/MS	-	0.01	0.033	-	0.012	0.04	-	-	-	-	-	-
Concejero et al. [43]	2014	SA	UPLC-MS/MS	-	0.6	1.7	-	2	7	-	-	-	-	-	-
Bonnafoux et al. [45]	2017	SIDA	UHPLC-MS/MS	-	-	-	-	-	-	*d_1/2_*-CG3SH ^d^	0.06	0.21	*d_1/2_*-GC3SH ^d^	0.18	0.61
Vanzo et al. [46]	2017	EC/ISC ^a,c^	HPLC-MS/MS	*d_6_*-C4MSP	0.09	0.28	*d_10_*-G4MSP	0.06	0.17	-	-	-	-	-	-
Tonidandel et al. [54]	2021	SIDA	UPLC-MS/MS	*d_3_*-C3SH	1.5	5	*d_3_*-G3SH	1.5	5	-	-	-	-	-	-

^a^ SIDA was used when the concentration of precursors was lower than the LOQ; ^b^ SIDA: stable isotope dilution assay, use of a stable-isotope-labeled internal standard, ISC: internal standard calibration, using an internal standard that is not an isotopologue of the target analyte, EC: external calibration, SA: Standard addition; ^c^ Although a stable-isotope-labeled internal standard was used, it was not an isotopologue of the target analyte; therefore, the method cannot be considered SIDA.

#### 3.2.2. Standard Addition

SA is an alternative method for the quantification of the target analyte, with an intrinsically matrix-matched calibration. Concejero et al. [43] utilized SA, spiking the samples with a known concentration of analytes, so each sample was analyzed twice and the difference in peak area was standardized by the known concentration added. Disadvantages of SA lie in the inefficiency of having to analyze each sample multiple times, as well as the increased error introduced by the repeated additions and analyses, and volume changes in the sample over time.

#### 3.2.3. Calibrations Using an Internal Standard

An alternative to EC is to use an IS and create a calibration curve relating the concentration of the analyte to the ratio of the analyte and IS responses [51]. An IS is used for most analytical methods for thiol precursors to account for the potential variation in sample preparation and instrumental performance. Unlike EC, where the area of the target analyte peak is directly compared with calibration curves to determine the concentration, ISC reduces the impact of the variation in instrumental performance or sample preparation between samples by assuming that the target analyte and IS are affected equally by any variation. An IS can be either an unlabeled or non-isotopomeric compound that does not naturally occur in the matrix, or an isotopologue of the compound of interest. Although the synthesis of a range of stable isotopically labeled analogues of most identified precursors to 3SH has been reported [29,34,40,45,55,56,57,58,59,60,61], the synthetic or financial requirements to obtain these ISs can prevent some researchers from accessing them. Methods that do not depend on SIDA have been developed and reported but may suffer from other limitations [43]. It is also worth noting that using an IS, whether an isotopologue of the analyte or simply a structurally related compound, produces a method with lower LOD and LOQ than methods relying on SA and no IS [43,46].

#### 3.2.4. Unlabeled IS (or Non-Isotopomeric IS)

An unlabeled IS can be a useful alternative to more expensive options (i.e., labeled isotopomeric analogues). For example, an unlabeled IS has been used to quantify dimethyl sulfide in wines when appropriate statistical techniques are employed, and the increases in LOD and uncertainty are offset by the lower cost and time [62]. Similar to EC, the matrix effects when using an unlabeled IS must be considered to account for any variance in the behavior of the IS and analyte during analysis. One way to account for this is to use multiple potential IS compounds and compare the performances of these to find which is optimal, such as choosing between isotopologues of G3SH and C3SH for CG3SH quantification [53].

#### 3.2.5. Labeled IS for SIDA

**Scheme 3:**
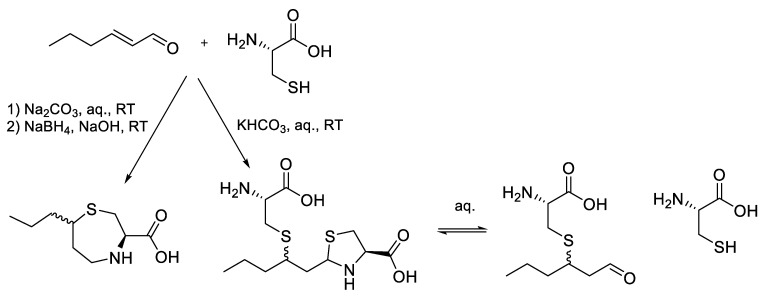
Products arising from the conjugate addition of Cysteine to *E*-2-hexenal under basic conditions—left per Starkenmann et al. [63], right per Wakabayashi et al. [64].

As mentioned previously, a stable, isotopically labeled version of the target analyte is the preferred IS, as it can be assumed that the two compounds are affected equally by any variation, with the same retention times, using a stable isotope dilution assay (SIDA). SIDA is advantageous when the measurement technique involves measuring by mass, as the analogues can be distinguished by their molecular weight, such as MS detectors. One of the earliest syntheses of C3SH was conducted by Tominaga et al.; however, the only characterization performed to assess purity was performed via the quantification of free and bound cysteine in the reaction products [22]. Subsequent attempts to reproduce this synthesis led to a mixture of reaction products, with C3SH being a minor component of this mixture (Scheme 3) [61,63,64].

**Scheme 4:**
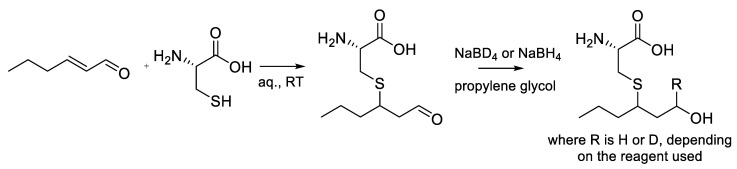
Proposed synthesis of Cys3SH and Cys3SH-*d_1_* by Tominaga et. al. [22] and Peyrot des Gachons et. al. [29], respectively.

The synthesis reported by Tominaga et al. [22] was also used to form an isotopologue of C3SH using sodium borodeuteride instead of sodium borohydride to form C3SH-*d_1_*, although NMR data were also not reported for this compound (Scheme 4) [29]. This reduction with sodium borodeuteride has proved challenging for other researchers, even under strictly aprotic conditions, such as using dry glassware and *d_1_*-ethanol (EtOD) as the solvent, with Roland et al. [52] reporting a mixture of products, some deuterated and some hydrogenated, in their synthesis of deuterium-labeled G3SH.

**Scheme 5:**
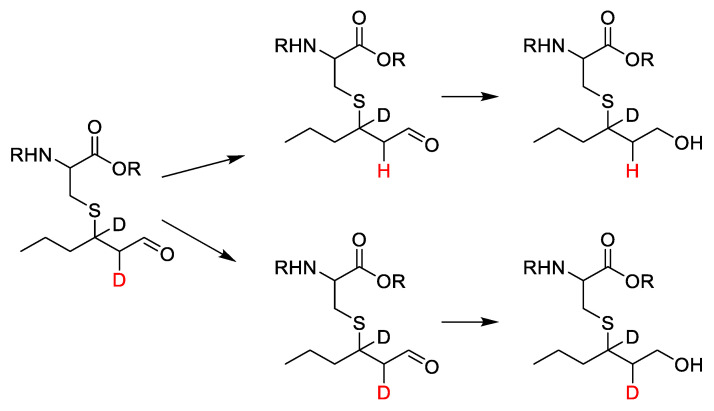
Illustration of the issues faced by Bonnafoux et al. [45,66]. While in the aldehyde form, keto-enol tautomerism leads to incomplete loss of deuterium from C-2. Once reduced to alcohol, no further tautomerism can occur and the substituents are finalized.

Indeed, even if the synthesis had worked as proposed, and C3SH-*d_1_* was obtained, this would not be an ideal IS as there is only one mass unit difference between the standard and the target analyte. While it is generally understood that higher degrees of labeling are preferred, it has been observed that, when the IS has many deuterium atoms incorporated, the isotope effects this causes can have a detrimental effect on the IS’s performance. For example, deuterium interacts differently, albeit subtly, with the stationary phase of HPLC systems, causing the deuterium-labeled isotopomer to elute very slightly earlier than the natural compound [65]. When the number of deuterium labels increases, this effect is also increased. In complex matrices, the slight shift in the retention time between the target analyte and the isotopomer can lead to different compounds co-eluting with the analyte and IS, which may cause significant differences in the ionization efficiency between the analyte and IS [51,65]. The appropriate number of isotopic labels in the IS to achieve appropriate mass separation between the target analyte and the isotopologue is dependent on the method. ^13^C and ^15^N-labeled isotopologues do not tend to suffer from isotope effects to the same extent as deuterium-labeled isotopologues [51]. With this in mind, a useful approach to the synthesis of isotopomers of thiol precursors may be to incorporate ^15^N into the amino acid moiety of the molecule. Indeed, this was the approach taken for one of the earliest syntheses of an isotopomer of C3SH by Murat et al. [34]. However, the resulting standard, ^15^N-C3SH, had the same issue as C3SH-*d_1_*, being only one mass unit away from the target analyte. As the number of sites that can be labeled with ^15^N in thiol precursors is limited, this is likely inviable for future labeling studies. Even when sufficient labels are incorporated into the compound, the position of these labels is important. Bonnafoux et al. [45,66] encountered some issues due to keto-enol tautomerism, leading to the loss of deuterium labels on the C-2 of the hexanol moiety of 3SH precursors. This resulted in a mixture of *d*_1_- and *d*_2_-precursors, which are difficult to use for quantification (Scheme 5).

### 3.3. Concluding Thoughts on 3SH Precursor Method Development

Ultimately, all analytical methods discussed here are suitable for use in oenological samples, as the concentration of the precursors being studied is high enough that sensitivity is not an issue. However, it is also important to consider that the LOD/LOQ is not the ultimate measure of method quality, as some methods, such as EC, may be hampered by matrix effects that are not present in the calibration matrix, affecting the accuracy of the measurements. Despite these issues, and other challenges associated with LC-MS analysis, LC-MS has rapidly become the primary technique for the analysis of 3SH precursors in oenological matrices.

## 4. Further Exploration of the Pathways to 3SH

**Scheme 6:**
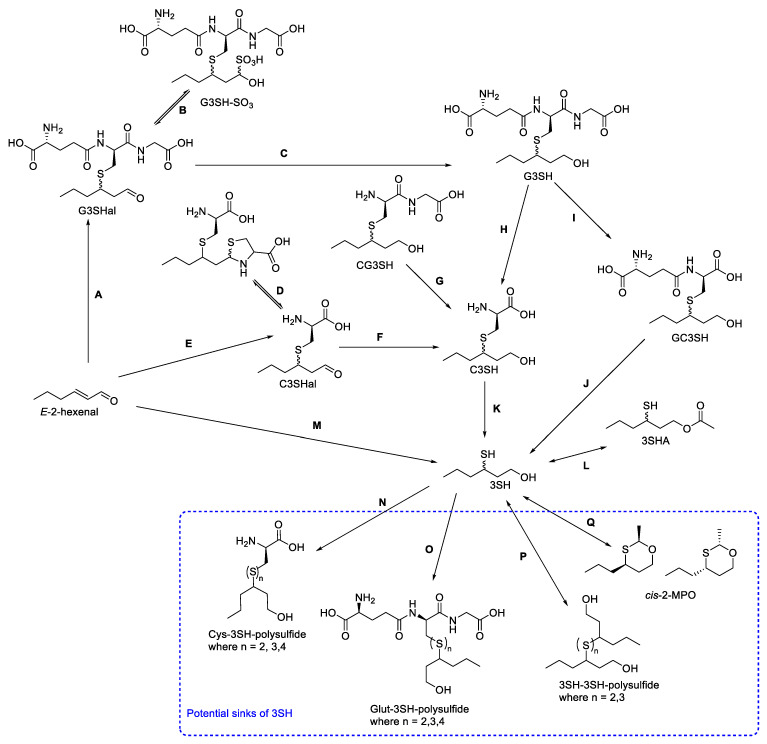
Summary of the current understanding of 3SH formation in enological matrices, as well as key potential sinks of 3SH. Adapted from: A: [69], B: [44,69], C: [70], D: [69], E: [69], F: [69]; G: [66], H: [55], I: [66], J: [66], K: [22,71], L: [72], M: [73], N: [74], O: [74], P: [74], and Q: [75].

Researchers have used the above analytical methods to widely explore the biogenesis of the volatile thiols 3SH and 3SHA in wines, with a focus on C3SH and G3SH. However, our current understanding of this pathway from these precursors only explains ~50% of the 3SH produced in the finished wine [18,66]. Several studies have shown little correlation between the precursor content of the must and the thiol content of the finished wines [67]. The juice composition strongly influences the release of thiols from their precursors [68], and there are also alternative sources and sinks for 3SH, some confirmed, and others proposed (Scheme 6).

To investigate the precursors involved in 3SH formation, the analytical methods discussed previously have been applied to a number of studies investigating the metabolism of confirmed and putative 3SH precursors and the impact of the must composition, yeast strain, and fermentation conditions on the metabolism. Such studies have included both synthetic grape media and grape juice, which each have their own challenges. There is a limit to the extent of what can be learned through model systems, but controlled studies in real media are more challenging. In fact, even the amino acid profiles of synthetic grape media used in the literature have a strong influence on both the consumption of C3SH and G3SH, as well as on the release of 3SH and 3SHA [76].

Given the impact of many other components of grape juice on precursor consumption and thiol release [68,77], it is unsurprising that studies using grape juice are likely to provide the most insight into 3SH biogenesis. Many of the stable-isotope-labeled isotopomers of thiol precursors can be spiked into samples of real grape juice, either to confirm the nature of these compounds as precursors, or to assess the conversion in real samples. Spiking real grape juice with C3SH-*d_8_* allowed Subileau et al. [56] to gain important insight into the biogenesis of 3SH, in that neither C3SH nor *E*-2-hexenal were the major sources of 3SH under the fermentation conditions trialed. This is another situation where the location of the incorporation of the labels is important; if the labels can be lost during tautomerism or reaction with components within the wine matrix or are not retained when 3SH is cleaved from the rest of the precursor molecule, the isotopic labels cannot be tracked through to the finished thiols. Bonnafoux et al. [66] found that the potential loss of deuterium labeling during fermentation experiments resulted in an inability to quantify some of the thiols that were produced at extremely low levels, emphasizing the potential for labeling with stable isotopes other than deuterium. Additionally, spiking experiments with isotopologues of thiol precursors can impede the analysis of samples, requiring either an alternative IS or reliance on a different quantification method, such as SA [56,66].

Methods to identify key compounds in grape juice are key to the further exploration of the biogenesis of 3SH—the existence of G3SH as a key precursor to 3SH was hypothesized but could not be confirmed until it was identified in must, and the fermentation of stable-isotope-labeled G3SH released the correspondingly labeled 3SH [52,56]. Further to this, the metabolism and conversion of precursors during fermentation have been explored under a variety of conditions, enabling the identification of additional precursors, as well as key metabolic pathways [39,70,78,79]. These studies of thiol biogenesis and precursor consumption have been thoroughly reviewed recently, and extensive discussion of these studies is outside of the scope of this work [18,20,21,80,81].

### 4.1. Aldehyde Conjugate Precursors

Fermentation spiking experiments with stable-isotope-labeled *E*-2-hexenal led to the initial identification of 3*S*-glutathionylhexanal (G3Shal) in oenological samples as the stable-isotope-labeled version [70]. The existence of this compound was predicted, as the chemical addition of sulfur-containing compounds to the 1-hexanol moiety can only occur for the α,β-unsaturated carbonyl compound, *E*-2-hexenal. Although the enzymatic addition of glutathione may occur, it has also been demonstrated that the chemical addition of glutathione to 2-hexenal (Scheme 6 step A) occurs in grape-juice-relevant conditions [69]. G3Shal has been identified in grape juice, and even post-fermentation in finished wines [44,69,82]. One characteristic of aldehydes is their tendency to react with bisulfite or sulfur dioxide to form the corresponding sulfonic acids [83,84,85]. In fact, it has long been suggested that most aldehyde-containing compounds found in wine exist in an equilibrium between the carbonyl and sulfonic acid forms (such as the equilibrium shown in step B of Scheme 6) [84]. As such, it is unsurprising that the first identification and isolation of G3Shal from grape juice also reported the presence of G3SH-SO_3_, the bisulfite adduct of G3Shal [44]. Thibon et al. [44] successfully synthesized these compounds and estimated their concentrations in grape juice via SA. However, the formal quantification of these precursors using a validated method has not yet been reported. As the aldehyde functional group behaves very differently to the alcohol functional group found in G3SH and C3SH, ideally, an aldehyde-containing IS should be used for quantification. The synthesis of an isotopically labeled IS, G3Shal-*d_8_*, has been reported [58], but the quantification of G3Shal in real samples still poses a challenge. G3Shal has also been shown to undergo tautomerism in wine-like solutions, which could give rise to additional precursors, but this requires further study [86].

Unlike G3Shal, the cysteine–hexenal conjugate, C3Shal, is yet to be identified in grape juice or wine, to the best of our knowledge. Under synthetic conditions in the work of Clark and Deed, the uncyclized form C3Shal was successfully formed through the chemical addition of cysteine to *E*-2-hexenal (Scheme 6 step E) [69]. However, this is in equilibrium with the cyclized form (Scheme 6 step D), which is the only product isolated when C3SHal is produced in pure synthetic studies [61,63,64,69]. It is hypothesized that this equilibrium in step D prevents the formation of the bisulfite adduct of C3Shal, unlike the behavior of G3SHal (Scheme 6 step B) [69].

The development of analytical methods specifically targeting the detection and quantification of these aldehyde conjugates will be another step forward in unraveling 3SH biogenesis during fermentation. With these tools, researchers would be able to better explore the left-hand side of Scheme 6, furthering our understanding of the link between *E*-2-hexenal and 3SH.

### 4.2. Dipeptide Thiol Precursors

While the mono- and tripeptide precursors to 3SH, G3SH, and C3SH have most commonly been investigated, the discovery that G3SH is converted to C3SH and 3SH during fermentation further suggested that the dipeptide S-conjugates CysGly-3SH and γ-GluCys-3SH (CG3SH and GC3SH) may also be produced from G3SH [55]. However, initial analytical conditions used by Grant-Preece et al. [55] did not permit the detection of these compounds in any of the samples analyzed. When conclusively identified and quantified in wine samples, CG3SH was found to be present at very low concentrations, up to 11 µgL^−1^, suggesting that it is a short-lived intermediate between G3SH and C3SH when G3SH is metabolized [53]. Indeed, the low concentration of CG3SH is likely the reason Peyrot des Gachons et al. [31] was unable to detect CG3SH in wine samples unless they were first treated with γ-glutamyltranspeptidase. GC3SH has since also been identified and quantified in Sauvignon blanc juice, and recent work has also demonstrated the distinct roles of GC3SH and CG3SH (Scheme 2) by spiking natural must with deuterated precursors [45,66]. While CG3SH is metabolized to form C3SH (Scheme 6 step G), the metabolism of GC3SH did not produce C3SH and instead produced free 3SH (Scheme 6 step J), albeit with a very low conversion yield [66]. Additionally, it has been shown that, while G3SH is metabolized to GC3SH (Scheme 6 step I), the same cannot be said for CG3SH [66].

The similarity of these dipeptide precursors to C3SH and G3SH means that new methods should not need to be developed specially to detect them. Many groups have successfully incorporated dipeptide precursor analysis into their existing thiol precursor methods with little difficulty [39,45,53,79]. However, the naturally low concentrations of these compounds in juice and wine make their detection challenging, especially when no preconcentration steps are employed.

### 4.3. Sinks of 3SH and 3SHA

The potential sinks of 3SH all arise from the reactivity of thiols with other components of the wine matrix, including thiol-containing compounds, and acetaldehyde [74,75,87]. The reaction of thiols with other thiol-containing compounds, such as glutathione and cysteine, as well as 3SH and 3SHA, forms polysulfides (Scheme 6 steps N, O, and P), and this reaction can be reversible under some conditions [74,87]. The release of 3SH from polysulfide forms in wine matrices has not yet been confirmed. In an oenological matrix, the abundance of acetaldehyde can also lead to the formation of the volatile compound *cis*-2-methyl-4-propyl-1,3-oxathiane (*cis*-2MPO) (Scheme 6, step Q), which can also be a source of 3SH if the reverse reaction occurs [75].

Further work is needed to elucidate the role of these sinks in depleting, or supplementing, the 3SH and 3SHA concentrations under oenological conditions.

## 5. A New Era of Thiol Exploration

The future of thiol precursor and volatile thiol analysis involves the development of new methods that are moving away from the traditional precursor analysis format. The QuEChERS (quick, easy, cheap, effective, rugged, and safe) method has become popular in the wider analytical community, particularly with pesticide residue analysis, as a practical alternative for the separation of compounds by polarity, without the requirement for SPE or niche consumables [88]. However, the application of this to the wine matrix has been limited until now. The recent work of Tonidandel et al. [54] has unlocked a new era for the exploration of thiol biogenesis by combining the analysis of common thiol precursors, C3SH and G3SH, with the analysis of the volatile thiols, 3SH and 3SHA, into a single protocol. This method has great potential to expand our understanding of the relationship between 3SH and 3SHA, and their precursors. The reduction in barriers to analyses makes the research into varietal thiols far more accessible, requiring only common consumables and access to an LC-MS/MS system, and should increase the range of studies carried out. Indeed, as our understanding of 3SH biogenesis grows, there is potential to add further precursors and even sinks of 3SH to this method, making it even more versatile. Only time will tell how this simplified and integrated method will enable researchers to further explore the biogenesis of thiols in juice and wine.

As new analytical methods for thiols and their precursors become more accessible and reliable, we can look forward to the further elucidation of this complex system impacting the aromatic profile of many wine styles.

## Figures and Tables

**Table 1:** A summary of early methods for the quantification of C3SH, comparing LOD and LOQ for methods using GC-MS and LC-MS.

Authors	Year	Instrument	Column Sorbent	Sample Volume	LOD (ppb)	LOQ (ppb)
Thibon et al. [35]	2008	GC-MS	35% Phenyl polysilphenylene-siloxane	0.5 mL	0.33	1.1
Luisier et al. [37]	2008	HPLC-APCI-MS	C18	8 mL	3	10
Thibon et al. [38]	2010	GC-MS	35% Phenyl polysilphenylene-siloxane	0.5 mL	0.0221	0.0884
Capone et al. [40]	2010	HPLC-MS/MS	C18	10 mL	0.04	0.12
Kobayashi et al. [39]	2010	HPLC-MS/MS	C18	5 mL	0.044264	0.132792
Roland et al. [41]	2010	NanoLC-MS/MS	C18	1.2 mL	0.309848	1.05127

**Table 2:** Summary of sample preparation and methods for the analysis of thiol precursors in oenologically relevant samples.

Authors	Year	Compounds Analyzed	Sample Matrix	Sample Volume	Sample Preparation	Derivatization	Instrument	Column Sorbent
Peyrot des Gachons et al. [29]	2000	C3SH	Sauvignon blanc juice	20 mL	pH adjusted to 8 in phosphate buffer, purified on weak cation exchange column, and unretained fraction percolated on a tryptophanase column before the analysis of the released thiols	None	GC-MS	Polyethylene glycol WAX
Luisier et al. [37]	2008	C3SH	White juice and wine	8 mL	Supelclean^TM^ Envi-18 SPE, eluted with MeOH, and concentrated before injection	None	HPLC-APCI-NI-MS	C18
Thibon et al. [35]	2008	C3SH	Grape must	0.5 mL	pH adjusted to 10, percolated through immobilized Cu^2+^ column, eluted with 25 mM HCl, dried under vacuum at 70 °C, clarified in EtOH/acetone, dried, and heptafluoracylation derivatization conducted before analysis	Heptafluroacylation	GC-MS	35% Phenyl polysilphenylene-siloxane
Capone et al. [40]	2010	G3SH, C3SH, and CG3SH	White juice and wine	10 mL	SPE on Strata SDB-L, eluted with MeOH, and concentrated before injection	None	HPLC-MS/MS	C18
Kobayashi et al. [39]	2010	C3SH, G3SH, and CG3SH	Grape berry tissues, juice, wines		Berries extracted by maceration at room temperature for 16 h in 10% MeOH with 0.1% formic acid. Solution was filtered before analysis	None	HPLC-MS/MS	C18
Roland et al. [41]	2010	G3SH and C3SH	Grape juice	1.2 mL	Extracted on strong cation exchange resin, purified on a Sep-Pak C18 cartridge, and eluted with MeOH.	None	NanoLC-MS/MS	C18
Mattivi et al. [42]	2012	G3SH and C3SH	Juice and wine samples		Filtration and direct injection	None	UPLC-MS/MS	C18
Concejero et al. [43]	2014	G3SH and C3SH	Red and white grape juice	500 g grapes, pressed	Filtration and direct injection	None	UHPLC-MS/MS	C18
Thibon et al. [44]	2016	G3SHA/SO3	Sauvignon blanc juice	5L	Separation of compounds on C18 MPLC, lyophilized, filtered, and injected	None	UHPLC-FTMS	C18
Bonnafoux et al. [45]	2017	G3SH and C3SH, CG3SH and GC3SH	White juice and wine	1 mL	Filtration and direct injection	None	HPLC-MS/MS	C18
Vanzo et al. [46]	2017	G3SH and C3SH	Sauvignon blanc grape berry		Extraction into 100% MeOH at room temperature, centrifuged, and injected	None	UHPLC-MS/MS	C18

**Table 3:** Summary of the reported method sensitivities for thiol precursor analytical methods utilizing a range of sample preparation techniques (use of SPE indicated in bold): ^a^ Polymeric SPE sorbent, Strata SDB-L ^b^ Strong cation exchange (DOWEX 50WX4-100 ion exchange resin), followed by C18 sorbent (Sep-Pak).

Authors	Year	Instrument	Sample Preparation	C3SH		G3SH	
				LOD (ppb)	LOQ (ppb)	LOD (ppb)	LOQ (ppb)
Capone et al. [40]	2010	HPLC-MS/MS	**SPE ^a^**	0.04	0.12	0.13	0.39
Roland et al. [41]	2010	NanoLC-MS/MS	**SCX-SPE ^b^**	0.310	1.05	0.0611	0.203
Mattivi et al. [42]	2012	UPLC-MS/MS	Filter	0.01	0.033	0.012	0.04
Concejero et al. [43]	2014	UHPLC-MS/MS	Filter	0.6	1.7	2	7
Vanzo et al. [46]	2017	UHPLC-MS/MS	Extract and filter	0.09	0.28	0.06	0.17
Kobayashi et al. [39]	2010	HPLC-MS/MS	Extract and filter	0.0443	0.133	0.0407	0.122

## Data Availability

Data is contained within the article.
